# Supplementary material for: Polyserine–tau interactions modulate tau fibrillization
Source: J Biol Chem. 2025 Jul 25;301(9):110523. doi: 10.1016/j.jbc.2025.110523 (PMC12926022; doi:10.1016/j.jbc.2025.110523)
Supplement: Supporting Figures [file mmc1.pdf]

Polyserine-tau interactions modulate tau fibrillization

James Pratt[1], Kathleen McCann[2], Jeff Kuo[1], Roy Parker[1,2*]

[1] Department of Biochemistry, University of Colorado Boulder, CO, USA

[2] Howard Hughes Medical Institute, University of Colorado Boulder, CO, USA

*Corresponding author: Roy Parker

Email: roy.parker@colorado.edu

Supporting information:

Document S1: Figures S1-S6

Movies S1-S8, Related to figures 1, S2, and S3

Figure S1

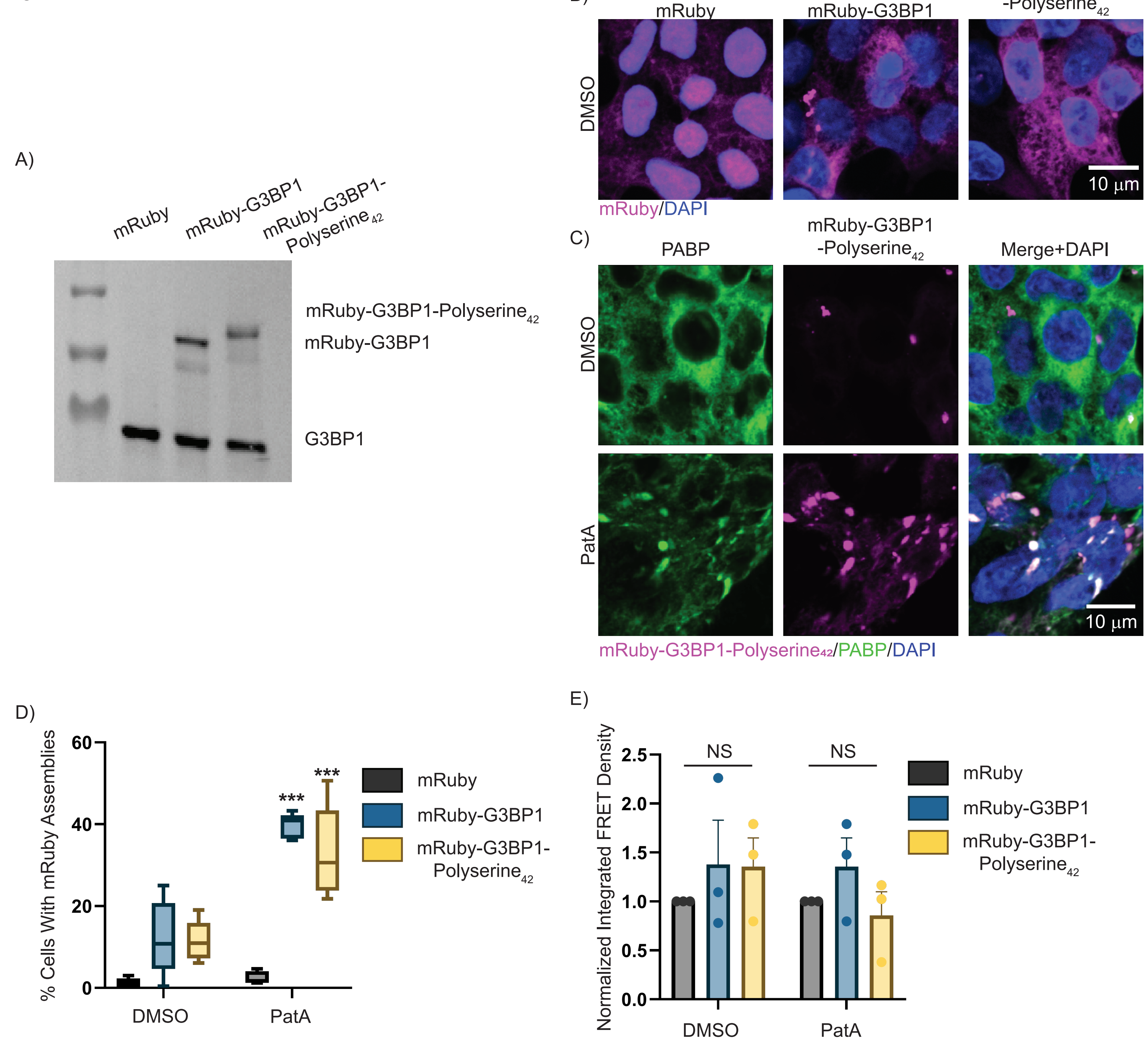
A)
mRuby
mRuby-G3BP1
mRuby-G3BP1-Polyserine42
mRuby-G3BP1-Polyserine42
mRuby-G3BP1
G3BP1
B)
mRuby
mRuby-G3BP1
mRuby-G3BP1 -Polyserine42
DMSO
10 µm
mRuby/DAPI
C)
PABP
mRuby-G3BP1 -Polyserine42
Merge+DAPI
DMSO
PatA
10 µm
mRuby-G3BP1-Polyserine42/PABP/DAPI
D)
% Cells With mRuby Assemblies
60
40
20
0
***
***
DMSO
PatA
mRuby
mRuby-G3BP1
mRuby-G3BP1-Polyserine42
E)
Normalized Integrated FRET Density
2.5
2.0
1.5
1.0
0.5
0.0
NS
NS
DMSO
PatA
mRuby
mRuby-G3BP1
mRuby-G3BP1-Polyserine42

<u>Figure S1</u>: Characterization and Validation of mRuby-G3BP1-Polyserine$_{42}$ Tau Biosensor Lines

(A) Western Blot stained with anti-G3BP1 conjugated to HRP from lysates prepared from HEK293T tau biosensor cells transduced with lentivirus expressing mRuby, mRuby-G3BP1, or mRuby-G3BP1-Polyserine$_{42}$. (B) Representative images of HEK293T tau biosensor cells stably expressing mRuby, mRuby-G3BP1, or mRuby-G3BP1-Polyserine$_{42}$ (magenta) fixed and nuclei stained with DAPI (blue). (C) Representative immunofluorescence images of HEK293T tau biosensor cells expressing mRuby-G3BP1-Polyserine$_{42}$ (magenta) treated with DMSO control (top) or PatA (bottom) for 12 hours. Cells were fixed and stained with anti-PABP (green), nuclei stained with DAPI (blue). (D) Quantification of percentage of cells with mRuby assemblies treated with DMSO as in (B). N = 15 images across 3 biological replicates. Statistics performed with Kruskal-Wallis test. All comparisons with DMSO are non-significant (**) P < 0.005. (E) Quantification of integrated FRET density (product of FRET+ cell percentage and median FRET intensity) normalized to DMSO/PatA mRuby control. N = 3 biological replicates. Statistics performed with Kruskal-Wallis. All comparisons are non-significant.

Figure S2

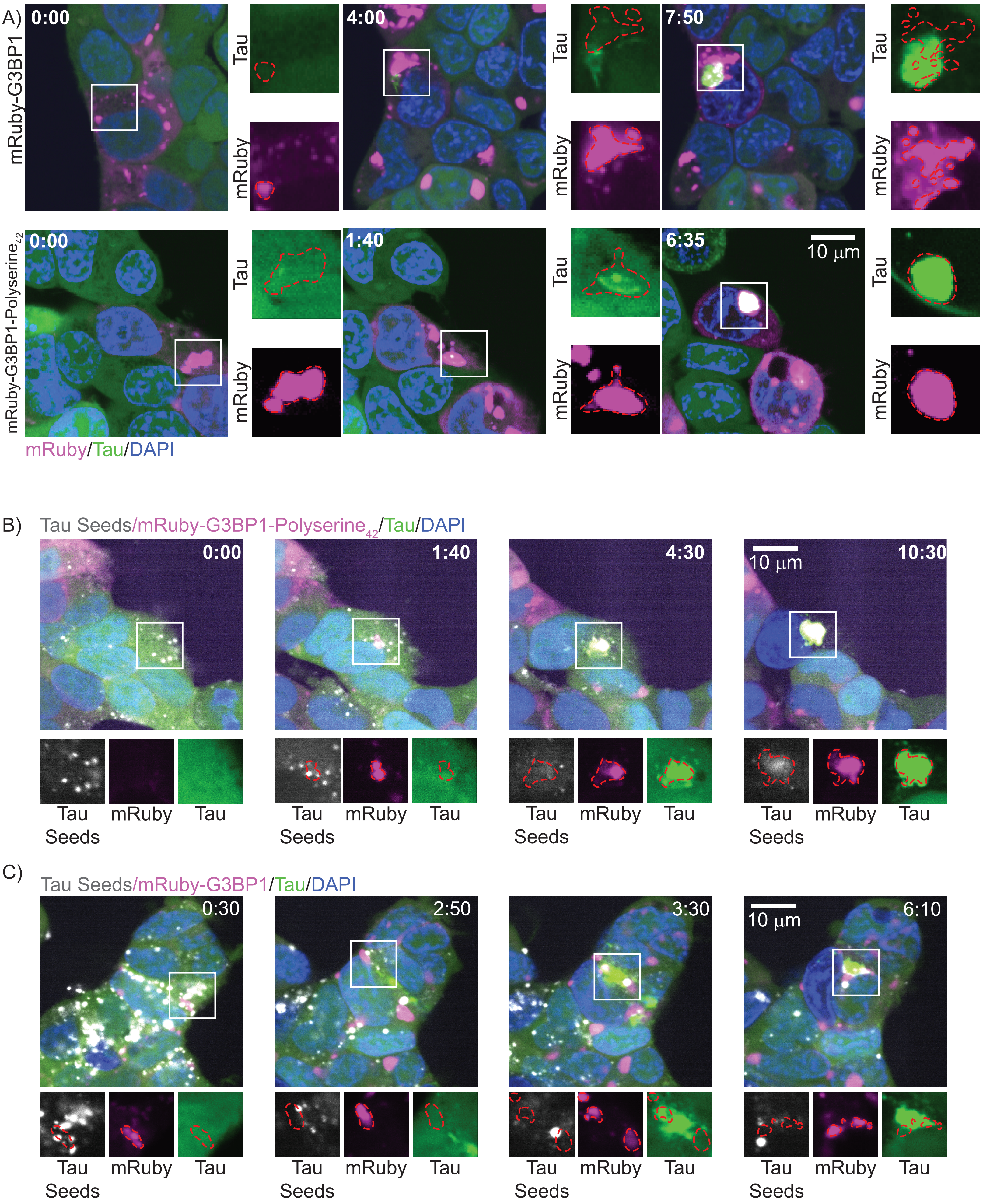

A)
mRuby-G3BP1
0:00
Tau
mRuby
4:00
Tau
mRuby
7:50
Tau
mRuby
mRuby-G3BP1-Polyserine42
0:00
Tau
mRuby
1:40
Tau
mRuby
6:35
10 μm
Tau
mRuby
mRuby/Tau/DAPI
B)
Tau Seeds/mRuby-G3BP1-Polyserine42/Tau/DAPI
0:00
1:40
4:30
10 μm
10:30
Tau Seeds
mRuby
Tau
C)
Tau Seeds/mRuby-G3BP1/Tau/DAPI
0:30
2:50
3:30
10 μm
6:10
Tau Seeds
mRuby
Tau

<u>Figure S2</u>: Live Cell Imaging of Tau Aggregation in mRuby-G3BP1 or mRuby-G3BP1-Polyserine$_{42}$ Expressing Tau Biosensor Cells

(A) Stills from live cell imaging of HEK293T tau biosensor cells expressing mRuby-G3BP1 (top panels, magenta) or mRuby-G3BP1-Polyserine$_{42}$ (bottom panels, magenta) transfected with tau brain homogenate from aged Tg2541 mice to induce endogenous tau aggregation (green), nuclei stained with Hoechst (blue). Time in hours:minutes post treatment with PatA and start of imaging. Channel breakouts of mRuby fluorescence and tau aggregates with mRuby assembly perimeter in red superimposed to all channels. (B) Stills from live cell imaging of HEK293T tau biosensor cells expressing mRuby-G3BP1-polyserine$_{42}$ (magenta) transfected with fluorescent tau fibers maleimide labeled with JF549 (grey) to induce endogenous tau aggregation (green), nuclei labeled with Hoechst (blue). Time in hours:minutes post treatment with PatA and start of imaging. Channel breakouts from each time point with mRuby assembly perimeter in red superimposed to other channels. (C) Stills from live cell imaging of HEK293T tau biosensor cells expressing mRuby-G3BP1 (magenta) transfected with fluorescent tau fibers maleimide labeled with JF549 (grey) to induce endogenous tau aggregation (green), nuclei labeled with Hoechst (blue). Time in hours:minutes post treatment with PatA and start of imaging. Channel breakouts from each time point with mRuby assembly perimeter in red superimposed to other channels.

Figure S3

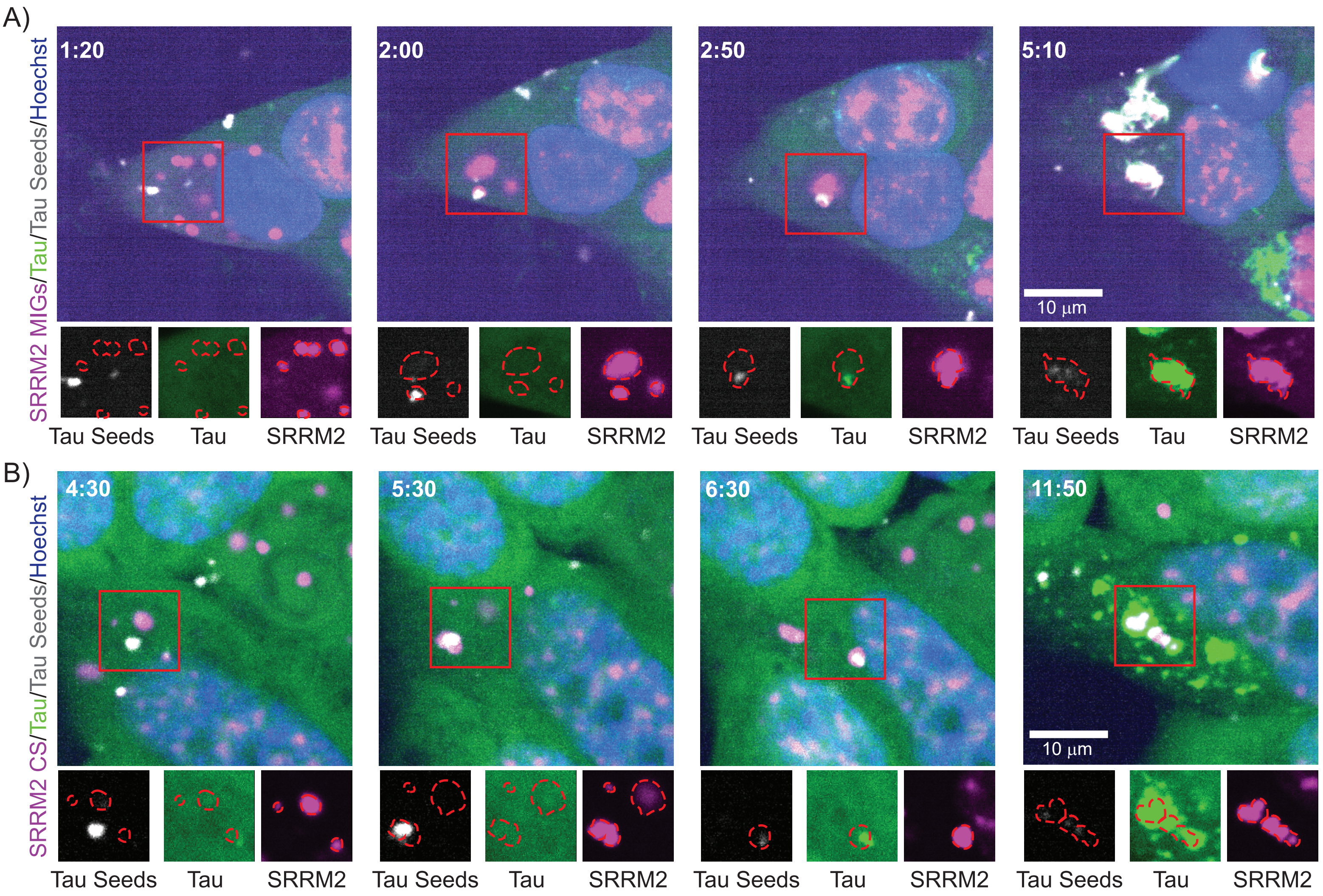
A)
SRRM2 MIGs/Tau/Tau Seeds/Hoechst
1:20
2:00
2:50
5:10
10 μm
Tau Seeds
Tau
SRRM2
B)
SRRM2 CS/Tau/Tau Seeds/Hoechst
4:30
5:30
6:30
11:50
10 μm
Tau Seeds
Tau
SRRM2

<u>Figure S3</u>: Live Cell Imaging of Tau Aggregation with Fluorescent Tau Seeds and SRRM2 Cytoplasmic Assemblies

(A) Stills from live cell imaging of HEK293T tau biosensor cells with endogenously tagged full length SRRM2-Halo labeled with JF549 (magenta) MIGs transfected with fluorescent tau seeds maleimide labeled with JF646 (grey) to induce endogenous tau aggregation (green), nuclei labeled with Hoechst (blue). Time in hours:minutes after transfection of tau seeds and start of imaging. Channel breakouts below each time point with SRRM2 assembly perimeter in red superimposed to other channels. (B) Stills from live cell imaging of cells as in (A) of SRRM2-Halo positive CSs.

Figure S4

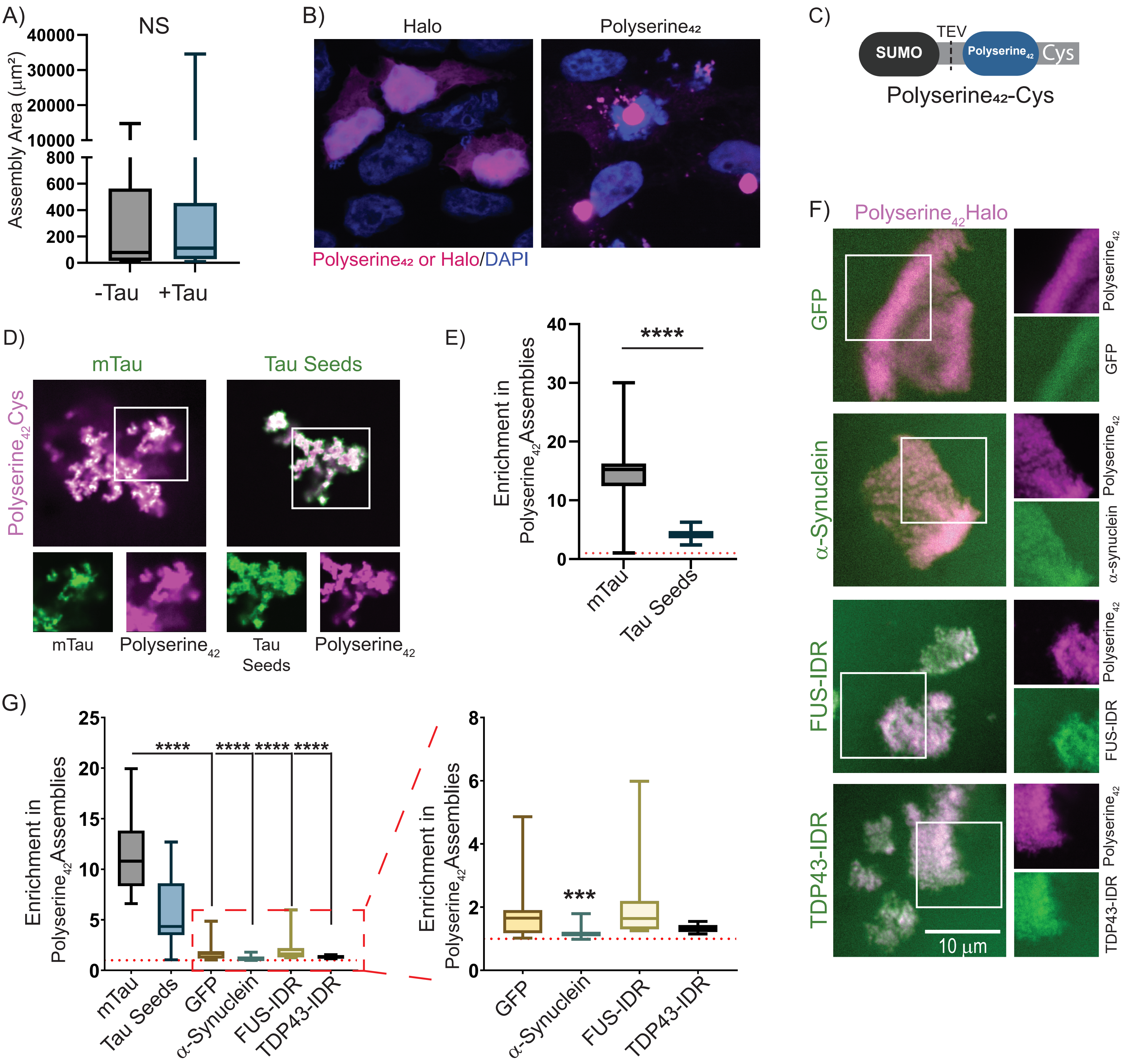
A)
NS
Assembly Area (μm²)
40000
30000
20000
10000
800
600
400
200
0
-Tau
+Tau
B)
Halo
Polyserine42
Polyserine42 or Halo/DAPI
C)
SUMO
TEV
Polyserine42
Cys
Polyserine42-Cys
D)
mTau
Tau Seeds
Polyserine42Cys
mTau
Polyserine42
Tau Seeds
Polyserine42
E)
Enrichment in Polyserine42Assemblies
40
30
20
10
0
****
mTau
Tau Seeds
F)
Polyserine42Halo
GFP
Polyserine42
GFP
α-Synuclein
Polyserine42
α-synuclein
FUS-IDR
Polyserine42
FUS-IDR
TDP43-IDR
Polyserine42
TDP43-IDR
10 μm
G)
Enrichment in Polyserine42Assemblies
25
20
15
10
5
0
**** **** **** ****
mTau
Tau Seeds
GFP
α-Synuclein
FUS-IDR
TDP43-IDR
Enrichment in Polyserine42Assemblies
8
6
4
2
0
***
GFP
α-Synuclein
FUS-IDR
TDP43-IDR

<u>Figure S4</u>: Polyserine Protein Interaction Specificity

(A) Quantification of assembly area of $Polyserine_{42}$-Halo with and without incubation with full length tau monomers. N = at least 807 assemblies across 3 biological replicates. Statistics performed with Kruskal-Wallis test with Dunn's multiple comparisons test. Comparison is nonsignificant. (B) Representative images of wildtype HEK293T cells transfected with a plasmid expressing $Polyserine_{42}$-Halo or Halo labeled with JF646 (magenta) for 48 hours. Cells were fixed and nuclei stained with DAPI (blue). (C) Schematic of recombinant $polyserine_{42}$-Cys. (D) Representative images of SUMO-$Polyserine_{42}$-Cys labeled with JF549-maleimide mixed with tau monomers or seeds maleimide labeled with JF646, channel breakouts are shown below each image (E) Quantification of protein enrichment in $Polyserine_{42}$ assemblies. N = at least 30 images across 3 biological replicates. Statistics performed with Kruskal-Wallis test compared to tau monomers. (***) $P < 0.001$ (****) $P < 0.0001$ (F) Representative images of $Polyserine_{42}$-Halo labeled with JF646 (magenta) mixed with GFP, or α-synuclein, FUS-IDR, or TDP-43 IDR each maleimide labeled with JF549 (green). Channel breakouts shown next to each image. (G) Quantification of enrichment of tau monomers, tau seeds, GFP, α-synuclein, FUS-IDR, or TDP-43-IDR in $Polyserine_{42}$ assemblies. Inset zoom shows the same data at a smaller y-axis scale. N = at least 30 images across 3 biological replicates, mTau and tau seeds data is the same as presented in figure 3C. Statistics performed with Kruskal-Wallis test compared to tau monomers. (***) $P < 0.001$ (****) $P < 0.0001$.

Figure S5

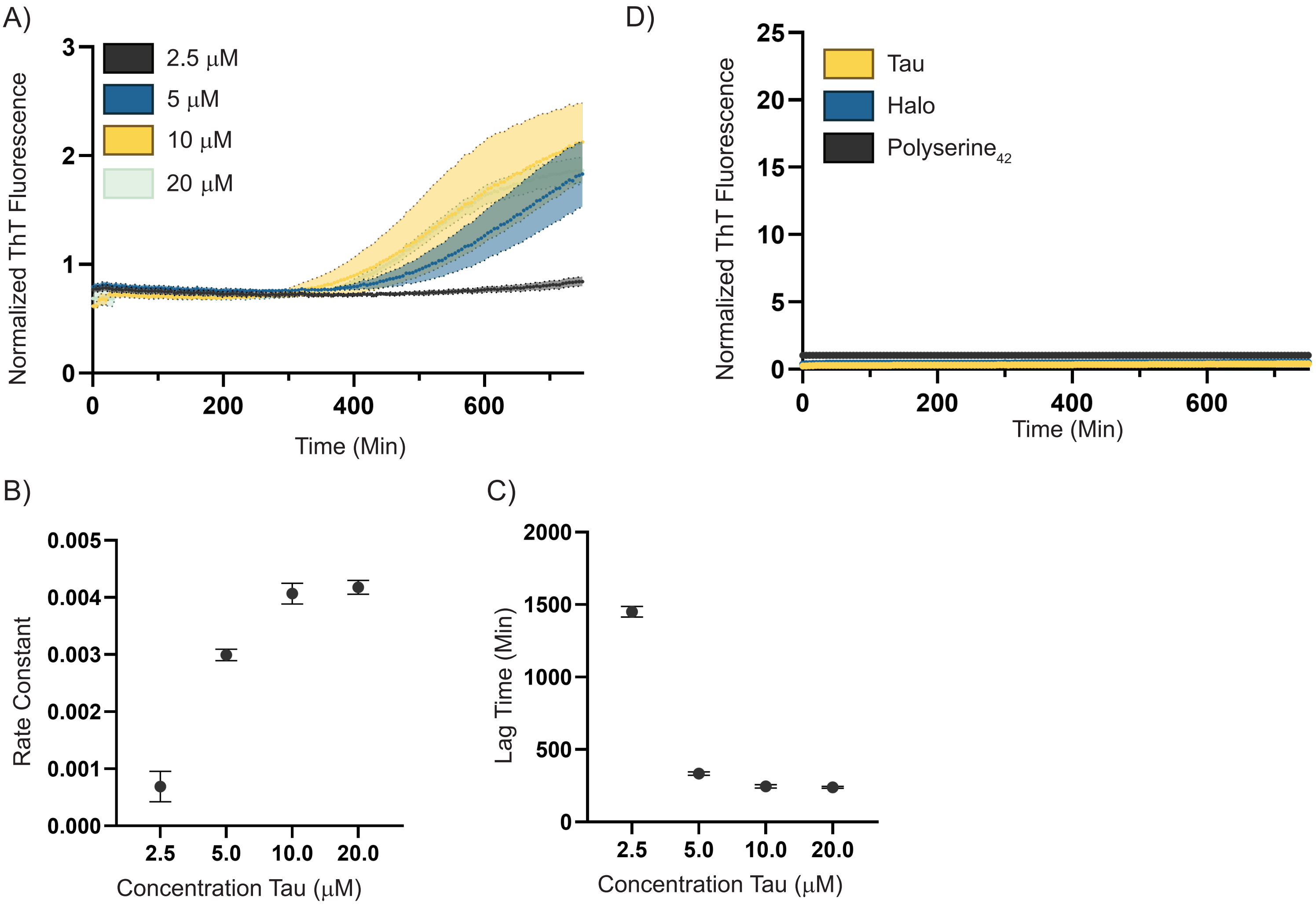

A)
Normalized ThT Fluorescence
2.5 μM
5 μM
10 μM
20 μM
Time (Min)
D)
Tau
Halo
Polyserine42
Normalized ThT Fluorescence
Time (Min)
B)
Rate Constant
Concentration Tau (μM)
C)
Lag Time (Min)
Concentration Tau (μM)

<u>Figure S5</u>: Thioflavin T Kinetics Optimization and Controls

(A) Increasing concentrations of tau were used in a ThT fluorescence assay, resulting curves are plotted over time with 2.5 μM (Black), 5 μM (Blue), 10 μM (Yellow) or 20 μM (Teal). Error bars are SEM from 3 technical replicates. (B) Logistic growth curves were fit to the data from A, rate constant of tau fibrilization was extracted and plotted against concentration of tau, error bars are SEM from 3 technical replicates. (C) Logistic growth curves were fit to the data from (A), lag time of tau fiber formation was plotted against concentration of tau, error bars are SEM from 3 technical replicates. (D) ThT fluorescence assay of tau with and without polyserine$_{42}$ or halo without polyU RNA, tau alone (yellow), tau+halo (blue), and tau+polyserine$_{42}$ (black). Error bars are SEM from 3 technical replicates.

Figure S6

A)

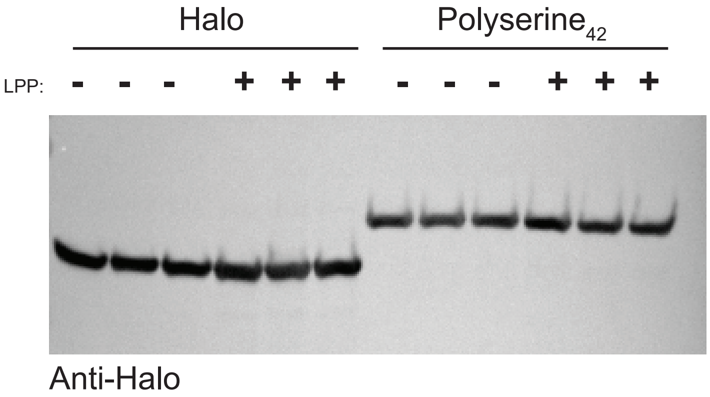

Figure S6: Phos-Tag Analysis of Halo or Polyserine$_{42}$-Halo From HEK293T Cells

(A) Plasmids expressing Halo or Polyserine$_{42}$-Halo were transfected into WT HEK293T cells. Cells were harvested after 48 hours, lysed with and without phosphatase inhibitor and treated with or without lambda-protein-phosphatase (LPP). Lysates were loaded onto a PhosTag SDS-PAGE gel, transferred to a nitrocellulose membrane which was probed with anti-Halo.

Movie S1: Live Cell Imaging of Tau Aggregation In Cells With mRuby-G3BP1 Stress Granules

Live cell imaging was performed using HEK293T tau biosensor cells stably expressing mRuby-G3BP1 (magenta) treated with PatA to form stress granules and transfected with tau seeds to form tau aggregates (green), nuclei labeled with Hoechst (blue). Images were collected every 10 minutes for 16 hours immediately after transfection of tau seeds.

Movie S2: Live Cell Imaging of Tau Aggregation in Cells With mRuby-G3BP1-Polyserine$_{42}$ Stress Granules

Live cell imaging was performed using HEK293T tau biosensor cells stably expressing mRuby-G3BP1-polyserine$_{42}$ (magenta) treated with PatA to form stress granules and transfected with tau seeds to form tau aggregates (green), nuclei labeled with Hoechst (blue). Images were collected every 10 minutes for 16 hours immediately after transfection of tau seeds.

Movie S3: Live Cell Imaging of Fluorescent Tau Seeds and Tau Aggregation in Cells With SRRM2-Halo Positive Mitotic Interchromatin Granules

Live cell imaging was performed using HEK293T tau biosensor cells stably expressing SRRM2-Halo visualized with JaneliaFluor 646 (magenta) transfected with fluorescent tau seeds maleimide labeled with JF549 (white) to form tau aggregates (green), nuclei stained with Hoechst (blue). Images were collected every 10 minutes for 16 hours immediately after transfection of tau seeds.

Movie S4: Live Cell Imaging of Fluorescent Tau Seeds and Tau Aggregation in Cells With SRRM2-Halo Positive Cytoplasmic Speckles

Live cell imaging was performed using HEK293T tau biosensor cells stably expressing SRRM20Halo visualized with JaneliaFluor 646 (magenta) transfected with fluorescent tau seeds maleimide labeled with JaneliaFluor 549 (white) to form tau aggregates (green), nuclei stained with Hoechst (blue). Images were collected every 10 minutes for 16 hours immediately after transfection of tau seeds.

<u>Movie S5</u>: Live Cell Imaging of Tau Aggregation in Cells With PNN-Halo Positive Mitotic Interchromatin Granules

Live cell imaging was performed using HEK293T tau biosensor cells stably expressing PNN-Halo visualized with JaneliaFluor 646 (magenta) transfected with fluorescent tau seeds maleimide labeled with JF549 (white) to form tau aggregates (green), nuclei stained with Hoechst (blue). Images were collected every 10 minutes for 16 hours immediately after transfection of tau seeds.

<u>Movie S6</u>: Live Cell Imaging of Tau Aggregation in Cells With PNN-Halo Positive Cytoplasmic Speckles

Live cell imaging was performed using HEK293T tau biosensor cells stably expressing PNN-Halo visualized with JaneliaFluor 646 (magenta) transfected with fluorescent tau seeds maleimide labeled with JF549 (white) to form tau aggregates (green), nuclei stained with Hoechst (blue). Images were collected every 10 minutes for 16 hours immediately after transfection of tau seeds.

<u>Movie S7</u>: Live Cell Imaging of Fluorescent Tau Seeds and Tau Aggregation in Cells With mRuby-G3BP1-Polyserine$_{42}$ Stress Granules

Live cell imaging was performed using HEK293T tau biosensor cells stably expressing mRuby-G3BP1-polyserine$_{42}$ (magenta) treated with PatA to form stress granules and transfected with fluorescent tau seeds maleimide labeled with Janelia Fluor 549 (white) to form tau aggregates (green), nuclei stained with Hoechst (blue). Images were collected every 10 minutes for 16 hours immediately after transfection of tau seeds.

<u>Movie S8</u>: Live Cell Imaging of Fluorescent Tau Seeds and Tau Aggregation in Cells With mRuby-G3BP1 Stress Granules

Live cell imaging was performed using HEK293T tau biosensor cells stably expressing mRuby-G3BP1 (magenta) treated with PatA to form stress granules and transfected with fluorescent tau seeds maleimide labeled with Janelia Fluor 549 (white) to form tau aggregates (green), nuclei stained with Hoechst (blue). Images were collected every 10 minutes for 16 hours immediately after transfection of tau seeds.
